# Notes and News

**Published:** 1887-11-19

**Authors:** 


### NOTES AND NEWS.

COLLECTED AND COMMUNICATED.

THE spectroscope, in itself one of the most fascinating inventions of modern science, has begun to afford valuable evidence in cases of poisoning. In a recent case of sudden death no definite cause could be assigned, when, by the aid of the spectroscope, bands of hæmatin were discovered in the patient's remains. This gave a clue, which subsequently led to the discovery that he had committed suicide by taking chlorate of potash. It transpired that his life was insured in a company that did not pay in cases of suicide, and he had doubtless thought that the poison he employed to take away his life would not be discovered, and that the insurance would be paid to his heirs. But for the employment of the spectroscope the terrible ruse would have succeeded.

THE Bishop of Liverpool, the Right Rev. Dr. Ryle, preached on the occasion of the seventy-eighth anniversary of the Derbyshire General Infirmary. In the course of his sermon the Bishop said: "He would try once more, as their preacher for the third time, to point out some lessons which they would do well to learn that day. In the first place, he asked them to observe how much there was about the human body in Bible religion. Some of the schools of heathen philosophers regarded the body with contempt, as a hindrance, and not a help—a clog and a drag, and not an aid to the soul. Let them turn to the Christian religion and mark what a contrast is presented. Whether they looked at its leading facts or doctrines, or practical instruction for the present, or hopes for the future, the human body was continually brought to the front, and its importance magnified. It is," continued his Lordship, "the highest wisdom both in the Church and the State never to forget the importance of the body. To promote cleanliness and temperance and social purity, to aim at the highest standard of sanitary arrangements, to encourage every movement which can increase health and longevity of a people; to provide as far as possible good air, good water, good dwellings, and cheap food, for every man, woman, and child in the land; these are objects which deserve the best attention, both of the Christian and the man of the world. There is a mine of deep truth in the saying, *Sanitas sanitatum omnia sanitas.*"

THE entrance scholarship of £125 of St. George's Hospital Medical School, open to the sons of medical men, has been awarded to Mr. R. G. Turner of the University College, Liverpool; the Holt Scholarships, value £100 each, have been awarded to Mr. W. Thelwell Thomas and Mr. Randal Lee.

THE £100,000 left to St. George's Hospital will not only tend to place it in easy circumstances, but also to the extent of the interest accruing from such a sum will be felt throughout the whole of the metropolitan hospital system. The demands made by St. George's on the Hospital Sunday Fund and on public subscriptions will be to a certain extent diminished, and there will of course result a corresponding increase to all the other participant charities. So true it is that the advantage of one hospital is the profit of all.

THE dispute about the position of the Sheffield Small-pox Hospital has been settled by the appointment of an experienced medical man, Mr. Shirley Murphy, who has reported against the continuance of the hospital on its present site, because of its dangerous proximity to dwelling houses. Mr. Justice Stirling has, in consequence, ordered it to be closed by the 15th prox. This is a new and, on the whole, we think, a wise departure. It is desirable that the judge should be fortified by medical authority in these cases.

A DECREASE all round is the report which comes from Liverpool concerning the Hospital Sunday Fund, the Saturday Fund, and the amount of donations contributed separately in connexion therewith. It is possible that in many cases the diminution reported in these collections is due to a want of energy and tact on the part of those who have the funds in hand. Too often the movements are promoted in a perfunctory and unenthusiastic manner. It should never be forgotten that where popular sympathy is needed to be evoked, energy, enthusiasm, and tact must be brought to bear upon the work in hand.

THE dispute at Oswestry about the cottage hospital is still actively continued. "Caritas," writing to the *Oswestry Advertiser*, considers that "the only remedy is that of cutting short the supplies." This, he says, has now commenced. The workmen of one of the largest works in the neighbourhood, who have been contributing out of their wages five or six shillings per week, have ceased to do so, and others, it is said, are preparing to follow.

THE attention which is now directed to M. Pasteur should bring to the mind the story of another believer in the remedial power of inoculation, who made his experiment, not *in corpore vili*, but on himself. In 1816, Dr. Eusebio Valli, an Italian physician, went to Havana to study the phenomena of yellow fever. There he determined to inoculate himself with the poisonous bile taken from the body of a patient who had died of the disease, and did so in the presence of two doctors of the town. His intention was to try the same remedies which he had already successfully used in dealing with the plague in the East. The characteristic symptoms of yellow fever soon appeared, but the means Valli employed for their relief failed of effect, and he succumbed to the disease within three days. The name of this martyr to medical science is almost forgotten now; though quite recently Dr. Freire, of Rio de Janeiro, has again taken up the idea of inoculation as preventive of yellow fever. One can only regret that Dr. Valli did not make his initial experiment on some inferior animal, and so test the value of his theory at a less cost than that of his own life. Thoughtful and intelligent physicians are not so numerous that they should be sacrificed to scientific research, even when they risk death of their own free will.

The following vacancies are announced this week, the amount of salary in each case being given after the name of the institution :—

**Resident Medical Officers.**

Bolton Infirmary. Junior House Surgeon.—£120 to £150.
Boscombe Provident Infirmary, Bournemouth. Resident Officer. —£60.
Hangleton Hospital, Hove. Medical Superintendent.—£50.
Lincoln County Hospital. House Surgeon.—£100.
Liverpool Dispensaries. Assistant Surgeon.—£80.
Royal Albert Idiot Asylum, Lancaster. Assistant Medical Officer. —£120 to £150.
Wallasey Dispensary. House Surgeon.—£120.

**Non-resident Medical Officers.**

Sheffield Medical Officer of Health.—£500.
National Hospital for Paralyzed and Epileptic, Bloomsbury. Physician for Out-Patients.
Parish of St. Mary, Islington. District Medical Officer.—£100.
Royal Westminster Ophthalmic Hospital. Assistant Surgeon.
Western General Dispensary. Honorary Surgeon.

**Matron.**

East Suffolk and Ipswich Hospital. Matron.—£60.

**Nurses.**

St. Saviour's Infirmary, East Dulwich.—£20 to £25.
Stafford Union. Trained Nurse.—£25.
Leicester Institution of Trained Nurses. Several. Apply, Lady Superintendent.
Burton-on-Trent Nursing Institution. Trained Nurse. Apply, Lady Superintendent.
Great Yarmouth Temporary Hospital. Night Nurse.—£20.
Nursing Institution, Burton-on-Trent. Surgical, Medical, and Midwifery training. Also Surgical and Medical.
Princess Alice Memorial Hospital, Eastbourne. Medical and Surgical.—£25.

---

The working men of Newcastle have subscribed liberally to the hospital during the last ten months. The annual subscriptions have now reached £4,049, as against £2,903 last year. It appears, however, that the contributions from the wealthier classes have not increased, and may even have diminished a little, in consequence of the abolition of the letter system. If the diminution be really occasioned by the withholding of letters, we cannot but express the conviction that it is highly discreditable to the wealthier classes of Newcastle.

---

"A gift of £50," says the *Somerset County Gazette*, "to the house surgeon of a county hospital, in recognition of twenty-one years' meritorious service, cannot be regarded as munificent, and probably every governor present at the annual meeting held at the Taunton and Somerset Hospital heard with pleasure a proposition that that sum should be doubled in favour of Mr. Rigdon on his resignation of the office. During his tenure of office Mr. Rigdon has earned the respect not only of the governors of the hospital, but also of all his fellow-citizens.

---

Dr. C. E. Fitzgerald, of Folkestone, points out, in a letter to the *Lancet*, the existence of a dangerous practice to which some of the mysterious outbreaks of fever which so often appear in schools and families may be due. It is that of stuffing mattresses and pillows with miscellaneous scraps of cloth which have not been cleansed before being employed in the manufacture of bedding. He was shown by a lady, who had had a number of bolsters and pillows unpicked in order that they might be cleaned, the materials with which they had been stuffed. These included scraps of dirty black serge, greasy silk, soldiers' coats, dirty worsted braid, soiled linen rags, coloured calico, and even walnut shells and pieces of crinoline wire. The bedding had been bought from an expensive and presumably respectable upholsterer; and the question arises whether he knew the contents of the pillows when he sold them, or whether he in turn was deceived by the manufacturer. In such a matter the purchaser is helpless. He cannot have every mattress or pillow he buys ripped up to see if it contains nothing but the hair or feathers of which it is said to be made. He must trust to the dealer's honesty, and this, it seems, is a broken reed.

The Governors of the Londonderry City and County Infirmary have resolved to consider the anomalous position of the Fever Hospital, which at present is under the same roof as the infirmary. A special meeting is to be called to discuss the propriety of removing the Fever Hospital to a distance. It is high time, not only that the Derry authorities, but all other hospital managers, should take steps to separate completely all infectious cases requiring hospital treatment from ordinary cases. The joining together of general and fever hospitals is a burlesque upon modern science.

---

The Mayor of Darwen (Mr. Thomas Grimm), on behalf of the organists, choirmasters, choirs, and other musical friends of Darwen, presented a beautiful pianoforte to the Blackburn and East Lancashire Infirmary on Saturday afternoon. Mr. Ashworth, as chairman of the House Committee, thanked his worship for the very valuable present, stating that the Board of Management accepted it in the spirit in which it was given, and was much pleased to hear of the kind feeling of the people of Darwen towards the institution. A concert was then given by several ladies and gentlemen, with Mr. Stockdale as accompanist.

---

The Blair Convalescent Hospital, which is a handsome and substantial structure, is now approaching completion. In a prominent position in one of the corridors there has been placed a slab which sets forth that "this convalescent hospital was built and endowed with funds bequeathed by the late Stephen Blair, Esq., of Mill Hall, Bolton-le-Moors, 1887." A well-qualified lady from the south of England has been appointed matron, and is now in residence at the lodge, pending the completion of her apartments in the new building. It is stated that twenty invalids will be received into the institution at the outset.

---

A curious protest on the part of Hospital Physicians and Surgeons appears in the Dublin *Evening News* in the form of a letter to the editor. The protest runs thus: "We, the undersigned, being members of the medical staff of the City of Dublin Hospital, having read in the report of the Mercers' Hospital inquiry a statement that in the City of Dublin Hospital there is a great amount of quarrelling going on, unanimously give an emphatic denial to the assertion.—Henry G. Croly; J. Hautrey Benson, M.D.; George F. Duffey, M.D.; W. J. Wheeler, M.D.; Henry Fitz Gibbon, M.D.; W. J. Smyley; Arthur H. Benton."

---

Familiarity may sometimes breed contempt, but it many also produces regard and esteem. The regard and esteem which we have for our contemporary the *Sanitary Engineer*, New York, leads us to regret that it has been found desirable to alter its title to the *Engineering and Building Record.* Of course the old name is still retained on the title, but only in the second place. No doubt the change of title has been found desirable on some grounds unknown to us, and the proprietors are the best judges of their own business. At the same time, English and European readers of the *Sanitar Engineer*, which has attained a position of international importance and value, should understand that the alteration of title means no retrogression editorially, but the contrary. To fearless outspokenness, honest criticism, painstaking elaboration of difficult problems, and sound practical advice by the ablest experts in their several professions, will now be added other and wider subjects of importance to engineers, architects, and sanitarians, as well as to the public at large. Those who do not yet know the *Engineering and Building Record*, and all who have an interest in sanitation, construction, engineering, and architecture, should purchase a copy at the London office, 92, Fleet Street, E.C., and judge for themselves. The chief of English sanitarians, when speaking of this journal a few months ago, said with truth: "It is by far the first and best of all professional papers devoted to the subjects which it has made its own."

As a "set off" to the foolish criticisms upon hospitals which recently appeared in the *Pall Mall Gazette*, the following, from one who signs himself "Ex-Patient," is worthy of consideration. He says: "I have had a little experience of hospitals in Scotland, and I can safely say that, as far as attention and nursing are concerned the Aberdeen Infirmary shows to advantage compared with any other. It is blessed and honoured by one professor at least—Professor Ogston—who brings with him an amount of skill, attention, and kindness of heart which is not practised by many in his position. His attention to the meanest street arab is as great as to the greatest."

In a year like the present, when the Hospital Saturday and Sunday Funds are suffering in many places, it is gratifying to be able to record that last week the Metropolitan Hospital Sunday Fund was found to have produced the largest amount ever raised by it for the London hospitals. This result has been due, not only to the devoted efforts of the eminent men who gave their time to attend and speak at public meetings, but to those of Mr. H. N. Custance, the Secretary and his small but capable staff of trained workers at the Mansion House. All the hospitals owe them grateful recognition for what they have done, as all will, we feel sure, most readily agree.

The voluntary principle of support will never, however, be successfully maintained if the London hospitals are not willing to co-operate to help themselves. This the more capable and intelligent managers who are responsible for the larger and best administered general and special hospitals have recognised cheerfully. It would be invidious for us to make a selection, though we have frequent inquiries from those who desire to give to the hospitals, but many a philanthropist will be glad to know and to note which of the hospitals have co-operated in the endeavour on self-help principles to awaken all classes to the duty of contributing to the hospitals on Hospital Sunday. There can, at least, be no doubt that anybody who sends a contribution to one of these co-operating institutions, may do so with the fullest confidence he is sending his money to be laid out to the best advantage for the relief of the sick and suffering. The following are the institutions, with the names of their representatives, who specially interested themselves in the work of arousing the people of London to a sense of the duty they owe to the hospitals:—The London Hospital, Mr. A. H. Haggard, secretary; the London Fever Hospital, the secretary; St. Mary's Hospital, Mr. P. Michelli, secretary; Middlesex Hospital, the secretary and assistant secretary; the Miller Hospital, Mr. W. J. Purver; the Royal Hospital for Children and Women, Mr. R. J. Kestin; St. George's Hospital, Mr. C. L. Todd; the City of London Lying-in Hospital, Mr. R. A. Owthwaite; the City of London Hospital for Diseases of the Chest, Capt. J. Storrar-Smith, secretary; Evelina Hospital for Children, Mr. T. S. Chapman; Queen Charlotte's Lying-in Hospital, Mr. T. Ryan; Victoria Hospital for Children, Capt. Blount, R.N., secretary; the Hampstead Dispensary, Mr. W. J. Fenn and Mr. G. Bushell, Mansion House. We trust each and all of these hospitals may receive some liberal donations before the year is out.

The Secretary of the Victoria Hospital for Children, Queen's Road, Chelsea (Captain W. C. Blount, R.N.), has issued a lithograph circular asking for advertisements to be inserted in the next Annual Report, to be issued in February, 1888, which, he states, is circulated amongst all the Royal Family, and numbers of influential people. He offers "a page advertisement, viz., insert bound in, both sides £10 10*s.*, one side £5 5*s.*" He adds, "to accept our offer will be conferring a benefit on this charity," and, he trusts, "considerable advantage to the advertiser." A postscript states that the Hôtel Metropole, Heath and Co., hatters, Mason and Co., Savory and Moore, Wright's Instruments, Belgrave Dairy Company, and Hutchings and Crowsley have taken space.

We shall be curious to see what the Governors think of this new departure on behalf of the Victoria Hospital for Children. We have heard of costless appeals—that is, appeals containing a sufficient number of advertisements to pay for the cost of their issue to the public; this is the first time, however, we have heard of a proposal to insert advertisements in the annual report of a hospital, and the experiment will be watched by hospital managers with some interest. There are no doubt other and better ways of raising from £50 to £100 for a hospital, the sum fixed in the present case where advertisements are limited to ten, each of a different character.

A daily contemporary, in calling attention to the legacy of £100,000 which was recently contributed to St. George's Hospital, takes the opportunity of inferring rather than stating that too large a sum is paid in wages and salaries for so small a hospital. This is really very unfair, because it is untrue. St. George's is not only not a small hospital, but the largest of all the voluntary hospitals excepting the London, and it stands at the head of them all for economical administration, the management expenses being only a little over five per cent. of the cost of maintenance. The hospitals seem to occupy the same position in the minds of some of our brother editors that the unemployed occupy in the minds of many worthy but thoughtless people: both need a little closer attention and more loving care at the hands of those who have it in their power to do, unintentionally, an infinity of harm, where so much encouragement and help is urgently required. If the public, on the one hand, would visit the workhouses and casual wards, and then insist on a little more humanity and a little less routine in the treatment of the poor, it would be to the no small advantage of the unemployed, and of themselves also. If the editors, on the other, would take the trouble to send a line of inquiry or a reporter to The Hospitals Association, Norfolk House, to find out whether a certain statement was justified by the facts or not, before criticising adversely a great charity like St. George's Hospital, then their views of hospital management would always be weighty, because they would never be wide of the facts. We wish that every editor's room could have a scroll suspended in a conspicuous place stating "Our hospitals require less criticism and more cash."

The paragraph in our contemporary was a particularly injurious one to St. George's Hospital, because it was not true in substance or in fact. Thus the average annual sum paid in salaries and wages during the last three years was less than £6,800, as compared with an average expenditure of £6,374 on the same account at the Middlesex Hospital. St. George's contains 351 beds, of which 269 are constantly occupied; the numbers in the case of Middlesex Hospital being 310 and 220 beds respectively. We have shown that the cost of management is smaller at St. George's than at any other voluntary hospital in London, and that comparison with the expenditure on salaries at the Middlesex—one of the very best managed hospitals in the country—proves that this item is equally moderate. The truth is, those who have not studied hospital administration have no idea of the cost of skilled nursing and clinical teaching. If they had they would give more to hospitals than they do at present.

